# Ethnic differences in the occurrence of acute coronary syndrome: results of the Malaysian National Cardiovascular Disease (NCVD) Database Registry (March 2006 - February 2010)

**DOI:** 10.1186/1471-2261-13-97

**Published:** 2013-11-06

**Authors:** Hou Tee Lu, Rusli Bin Nordin

**Affiliations:** 1 Clinical School Johor Bahru, Jeffrey Cheah School of Medicine and Health Sciences, Monash University Sunway campus, 8 Jalan Masjid Abu Bakar, 80100, Johor Bahru, Johor, Malaysia; 2 Department of Cardiology, Sultanah Aminah Hospital, Jalan Abu Bakar, 80100, Johor Bahru, Johor, Malaysia

## Abstract

**Background:**

The National Cardiovascular Disease (NCVD) Database Registry represents one of the first prospective, multi-center registries to treat and prevent coronary artery disease (CAD) in Malaysia. Since ethnicity is an important consideration in the occurrence of acute coronary syndrome (ACS) globally, therefore, we aimed to identify the role of ethnicity in the occurrence of ACS among high-risk groups in the Malaysian population.

**Methods:**

The NCVD involves more than 15 Ministry of Health (MOH) hospitals nationwide, universities and the National Heart Institute and enrolls patients presenting with ACS [ST-elevation myocardial infarction (STEMI), non-ST elevation myocardial infarction (NSTEMI) and unstable angina (UA)]. We analyzed ethnic differences across socio-demographic characteristics, hospital medications and invasive therapeutic procedures, treatment of STEMI and in-hospital clinical outcomes.

**Results:**

We enrolled 13,591 patients. The distribution of the NCVD population was as follows: 49.0% Malays, 22.5% Chinese, 23.1% Indians and 5.3% Others (representing other indigenous groups and non-Malaysian nationals). The mean age (SD) of ACS patients at presentation was 59.1 (12.0) years. More than 70% were males. A higher proportion of patients within each ethnic group had more than two coronary risk factors. Malays had higher body mass index (BMI). Chinese had highest rate of hypertension and hyperlipidemia. Indians had higher rate of diabetes mellitus (DM) and family history of premature CAD. Overall, more patients had STEMI than NSTEMI or UA among all ethnic groups. The use of aspirin was more than 94% among all ethnic groups. Utilization rates for elective and emergency percutaneous coronary intervention (PCI) and coronary artery bypass graft (CABG) were low among all ethnic groups. In STEMI, fibrinolysis (streptokinase) appeared to be the dominant treatment options (>70%) for all ethnic groups. In-hospital mortality rates for STEMI across ethnicity ranges from 8.1% to 10.1% (p = 0.35). Among NSTEMI/UA patients, the rate of in-hospital mortality ranges from 3.7% to 6.5% and Malays recorded the highest in-hospital mortality rate compared to other ethnic groups (p = 0.000). In binary multiple logistic regression analysis, differences across ethnicity in the age and sex-adjusted ORs for in-hospital mortality among STEMI patients was not significant; for NSTEMI/UA patients, Chinese [OR 0.71 (95% CI 0.55, 0.91)] and Indians [OR 0.57 (95% CI 0.43, 0.76)] showed significantly lower risk of in-hospital mortality compared to Malays (reference group).

**Conclusions:**

Risk factor profiles and ACS stratum were significantly different across ethnicity. Despite disparities in risk factors, clinical presentation, medical treatment and invasive management, ethnic differences in the risk of in-hospital mortality was not significant among STEMI patients. However, Chinese and Indians showed significantly lower risk of in-hospital mortality compared to Malays among NSTEMI and UA patients.

## Background

Acute coronary syndrome (ACS) encompasses a spectrum of clinical entities, ranging from unstable angina (UA), non-ST-segment elevation myocardial infarction (NSTEMI) to ST-elevation myocardial infarction (STEMI) [1]. In the Western world, ACS is the most common cause of death [2]. Cardiovascular disease (CVD) mortality is on the rise in the Asia Pacific countries (including Malaysia) that were undergoing rapid urbanization, industrialization and lifestyle changes [3]. According to the Global Burden of Disease Study (GBD), ischemic heart disease (IHD) is ranked first among the leading causes of mortality for eight regions in the world [4]. The World Health Organization (WHO) estimated that CAD will be the single largest cause of disease burden in many countries world-wide by the year 2020 [5]. Similarly, in Malaysia, CVD accounted for 147,843 admissions or about 6.91% of total admissions in Ministry of Health (MOH) hospitals in year 2009 [6]. CVD accounted for approximately 24.5% of death in government hospitals in year 2010 and is the leading cause of death in Malaysia [7].

Established coronary risk factors such as cigarette smoking, diabetes mellitus (DM), hypertension, obesity, sedentary life style and dyslipidemias still play major roles in CAD [8]. While conventional cardiovascular risk factors such as smoking, blood pressure and total cholesterol predict risk within these ethnic groups, they do not fully account for the differences in risk between ethnic groups, suggesting that alternative explanations might exist [9]. Epidemiological evidence that includes cross-sectional studies, coronary angiographic studies, and registry data showed significant differences between ethnic groups who were diagnosed with ACS in terms of presentations, risk factors, coronary vessel diameters, prognoses and outcomes [10-14].

With CVD accounting for most deaths globally, eliminating ethnic disparities in cardiac care has become a new challenge in the practice of cardiology [15]. Studies have shown that CVDs present differently in between ethnic groups [16,17]. Ethnicity has been shown to be an independent predictor of adverse cardiovascular outcomes in patients with atherothrombotic disease [18] and CAD [19].

ACS registries are important tools for analyzing disease management [20,21]. Exploration of registries data may lead to changes in disease management strategies and the national health care policies [22]. The National Cardiovascular Disease (NCVD) Database Registry is one of the pioneer projects to treat and prevent CAD in Malaysia. The project is a joint effort of doctors and nurses in public, private and academic medical institutions supported by the National Heart Association, National Heart Foundation, Clinical Research Centre and the MOH, Malaysia [23].

The aim of this study is, therefore, to identify the role of ethnicity in relation to the occurrence of ACS among high-risk groups in the Malaysian population using the Malaysian NCVD.

## Methods

A detailed description of NCVD has been reported elsewhere [23,24]. In brief, the NCVD for ACS is the first prospective, multi-center registry involving more than 15 MOH hospitals nationwide, universities and the National Heart Institute (IJN: *Institut Jantung Negara*). Since its establishment in 2006, all registry centers attempt to ensure the enrollment of an unbiased population. The NCVD is an observational prospective registry that collects data on ‘real-life’ ACS patients comprising STEMI, NSTEMI and UA according to demographic, epidemiological, management and outcome characteristics. The registry enrolls inpatients presented with ACS from January 2006 onwards and is still on-going at the time of writing.

Entry criteria of ACS include risk stratum of patients presenting with clinical features consistent with an ACS (chest pain or overwhelming shortness of breath) and defined by accompanying clinical, electrocardiographic and biochemical features. The final diagnosis of ACS was made by the attending physician using the following criteria: STEMI was diagnosed on the basis of the presence of acute chest pain with new or presumably new ST segment elevations more than 1 mm in two consecutive leads or the presence of a new left bundle branch block on the index or subsequent ECG with positive cardiac markers of necrosis [25]. NSTEMI was defined by ECG ST-segment depression or prominent T-wave inversion and/or positive biomarkers of necrosis in the absence of ST-segment elevation and in an appropriate clinical setting (chest discomfort or angina equivalent). UA was defined as angina pectoris (or equivalent type of ischemic discomfort) with any one of the three following features: a) angina occurring at rest and prolonged, usually more than 20 min; b) new-onset angina of at least Canadian Cardiovascular Society (CCS) classification III severity; c) recent acceleration of angina reflected by an increase in severity of at least one CCS class to at least CCS class III. The patient must also have normal cardiac biomarkers [26].

Demographic, significant risk factors or past medical history, anthropometric, ACS stratum, treatment, length of hospitalization, outcome (alive, dead) and complications (such as bleeding) data were obtained from the medical records and recorded on a standardized clinical research form by trained coordinators. Standardized definitions for all patient-related variables and clinical diagnoses were used [24]. Precise definition and quantification for risk factors, past medical history and ways of treatments were described in earlier publications [23,24].

**Figure 1:**
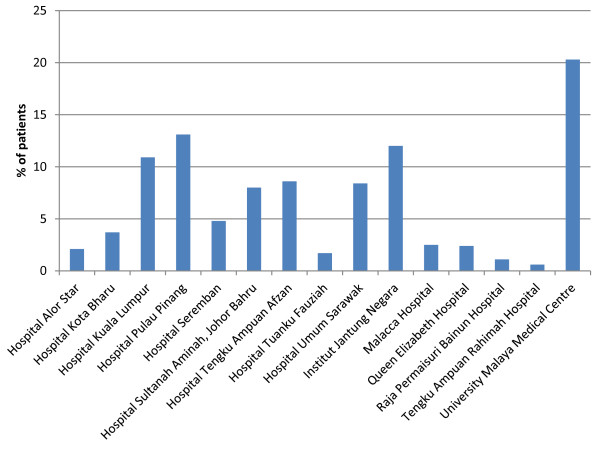
NCVD recruitment centres (N=13591).

Collected data were subsequently entered into a web-based centralized database with security password encryption according to individual centers. Regular data checks were performed and queries were generated for correction to ensure accuracy. Ethnicity that includes Malays, Chinese, Indians (major ethnic groups), Indigenous (Orang Asli), Kadazan, Melanau, Murut, Bajau, Bidayuh, Iban (minor ethnic groups) and other Malaysians were recorded. Ethnicity was self-reported and coded as mutually exclusive categories. We excluded 17 patients with missing ethnic information. A total of 13,591 patients were included in the analysis. The current study included ACS patients from March 2006 to February 2010 over a period of 4 years. All patients were enrolled in Malaysia at different centers as listed in Figure 1.

### Statistical analysis

Data were examined for normality distribution using the stem-and-leaf plot and Kolmogorov-Smirnov test. Descriptive statistics and baseline variables were presented as numbers and percentages, means and standard deviations, or medians and interquartile ranges. A chi square test was used to assess differences between categorical variables; a one-way ANOVA with post-hoc multiple comparisons (parametric analysis) or Kruskal-Wallis test (non-parametric analysis) was used to test differences across the four ethnic groups (Malays, Chinese, Indians, Others). For multivariate analysis, binary simple and multiple logistic regressions were used to model the dichotomous outcome of STEMI and NSTEMI/UA mortality among ethnic groups with adjustment for age and sex. We checked for significant interaction between age and sex and possible multicollinearity by examining the standard errors of the b coefficients. Any significant interaction should be included in the final model. Standard errors of the b coefficients that were less than 2 indicate the absence of multicollinearity. The results were reported as crude and adjusted odds ratio (OR) with 95% confidence interval for ethnicity. A p value of <0.05 was considered statistically significant. All statistical calculations were performed using the IBM SPSS Statistics 20.

### Ethics approval

This NCVD study was approved by the Medical Review & Ethics Committee (MREC), MOH Malaysia in 2007 (Approval Code: NMRR-07-20-250). MREC waived informed consent for NCVD.

## Results

### Socio-demographic characteristics

**Figure 2:**
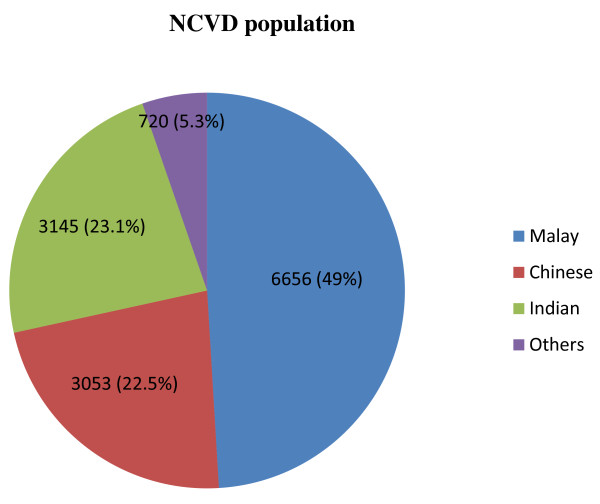
NVCD patients by ethnicity.

Socio-demographic characteristics and risk factors of the NCVD population are listed in Table 1. The distribution of the NCVD population was as follows: 49.0% Malays, 22.5% Chinese, 23.1% Indians, 5.3% Others (representing other indigenous groups and non-Malaysian nationals) and 0.1% missing data (Figure 2). The mean age of ACS patients at presentation was 59.1 (SD 12.0) years. More than 70% were males (p = 0.000). A large proportion of individuals within each ethnic group had more than two coronary risk factors including hypertension, DM and hyperlipidemia. Majority of patients were overweight [Body mass index (BMI) of more than 23 kg/m^2^] in accordance with the BMI for Asian populations [27]. Malays had the highest BMI (26.0 kg/m^2^) (p = 0.000). Indian had the biggest waist circumference (WC) (90.6 cm), the highest rate of DM (65.2%) and family history of premature CAD (22.2%) as compared to other ethnic groups (p = 0.000). Despite having the lowest BMI (24.8 kg/m^2^), Chinese had the highest rate of hypertension (76.1%) and dyslipidemia (51.8%) (p = 0.000). Others had the highest proportion of current or former smokers (65.7%) (p = 0.000).

**Table 1:** Socio-demographic characteristics and risk factors by ethnicity

**Socio-demographic characteristics & risk factors**				**Ethnic group**					**F**^**†**^	**χ**^**2‡**^	**df**^**#**^	**P**^**§**^
	**Malays**		**Chinese**		**Indians**		Others*					
Age (SD) (yr)	58.3	(11.5)	62.9	(12.1)	57.7	(11.9)	56.5	(13.0)	141.2			0.000
Men (%)	5230	(78.6)	2205	(72.2)	2275	(72.0)	579	(82.0)		88.7	3	0.000
Height (SD) (cm)	161.6	(8.2)	161.7	(7.6)	162.4	(9.0)	161.3	(8.3)	3.8			0.010
Weight (SD) (kg)	68.5	(14.5)	65.3	(12.2)	68.5	(14.6)	67.2	(13.6)	24.6			0.000
WC (SD) (cm)	88.9	(15.4)	86.9	(13.2)	90.6	(15.4)	86.2	(12.7)	17.0			0.000
BMI (SD) (kg/m^2^)	26.0	(4.4)	24.8	(3.7)	25.8	(4.5)	25.5	(4.1)	30.9			0.000
Smoking (%)	4141	(65.2)	1592	(55.1)	1430	(48.7)	439	(65.7)		259.0	3	0.000
Diabetes mellitus (%)	2646	(48.1)	1147	(45.0)	1780	(65.2)	200	(37.5)		315.9	3	0.000
Hypertension (%)	3859	(67.4)	2045	(76.1)	2010	(72.6)	400	(70.1)		73.9	3	0.000
Dyslipidemia (%)	2104	(48.4)	1144	(51.8)	1147	(51.2)	193	(42.6)		18.1	3	0.000
Family history of premature coronary artery disease (%)	725	(15.5)	235	(11.0)	454	(22.2)	69	(15.5)		101.1	3	0.000

*Others: Indigenous (Orang Asli), Kadazan, Melanau, Murut, Bajau, Bidayuh, Iban (minor ethnic groups) and other non-Malaysians.

^†^F statistics for one-way ANOVA.

^‡^Chi square value.

^#^Degree of freedom.

^§^P value.

WC: Waist Circumference.

BMI: Body Mass Index.

Note: We excluded 17 patients with missing ethnic information.

Table 2 shows the ethnic distribution of ACS and clearly indicated that Others (58.3%) had significantly higher STEMI (p = 0.000). Overall, more patients had STEMI than NSTEMI or UA among all the ethnic groups.

**Table 2:** Acute coronary syndrome by ethnicity

**ACS stratum**	**Ethnic group**				**χ**^**2‡**^	**df**^**#**^	**P**^**§**^
	**Malays**	**Chinese**	**Indians**	Others*			
STEMI**	3443(51.7)	1286(42.1)	1221(38.8)	420(58.3)	244	6	0.000
NSTEMI***	1762(26.5)	899(29.4)	972(30.9)	203(28.2)			
UA****	1451(21.8)	868(28.4)	952(30.3)	97(13.50)			

*Others: Indigenous (Orang Asli), Kadazan, Melanau, Murut, Bajau, Bidayuh, Iban (minor ethnic groups) and other non-Malaysians.

**STEMI: ST Elevation Myocardial Infarction.

***NSTEMI: Non-ST Elevation Myocardial Infarction.

****UA: Unstable Angina.

^‡^Chi square value.

^#^Degree of freedom.

^§^P value.

Table 3 shows that Malays had significantly higher mean total cholesterol (TC) and low density lipoprotein cholesterol (LDL) (5.5 mmol/l, 3.5 mmol/l, respectively) (p = 0.000). Indians had significantly higher mean fasting blood glucose (FBG) (8.4 mmol/l) (p = 0.000). Troponin T level was higher among Chinese (1.6 ng/ml). The level of CK is significantly higher in Others (1242 unit/l) (p = 0.000).

**Table 3:** Baseline investigation and clinical features on presentation by ethnicity

**Baseline investigation & presentation**	**Ethnic group**				**F**^**+**^	**P**^**#**^
	**Malays**	**Chinese**	**Indians**	Others*		
TC (mmol/l)	5.5(1.4)	5.1(1.2)	5.1(1.2)	5.3(1.3)	71.2	0.000
LDL (mmol/l)	3.5(1.3)	3.0(1.1)	3.2(1.2)	3.3(1.2)	93.7	0.000
HDL (mmol/l)	1.1(0.3)	1.2(0.4)	1.0(0.3)	1.1(0.3)	53.7	0.000
TG (mmol/l)	2.0(1.8)	2.0(1.2)	2.0(1.3)	2.0(1.1)	2.5	0.057
FBG (mmol/l)	8.0(3.7)	7.5(3.3)	8.4(3.9)	7.4(3.4)	34.7	0.000
Trop T (ng/ml)	1.5(4.6)	1.6(7.1)	1.2(3.6)	1.0(2.0)	1.2	0.300
CK (unit/l)	1075(1484)	884(1322)	789(126)	1242(1583)	36.3	0.000
Heart rate/min	83.1(21.1)	83.1(21.4)	85.9(23.1)	82.7(21.3)	14.1	0.000
SBP (mmHg)	138.9(28.9)	138.9(28.7)	140.2(27.9)	139.6(31.0)	1.8	0.153
DBP (mmHg)	80.9(16.5)	79.9(16.3)	82.2(16.0)	81.3(17.0)	9.7	0.000
Killip Class IV(%)^‡^	271(4.1)	100(3.3)	71(2.3)	28(3.9)	15	0.000
LVEF (%)^‡^	47(13)	46(13)	47(12)	47(13)	1.5	0.216

*Others: Indigenous (Orang Asli), Kadazan, Melanau, Murut, Bajau, Bidayuh, Iban (minor ethnic groups) and other Malaysians.

^+^Statistic for one-way ANOVA.

^‡^No.(%); all others: Mean (SD).

^#^P value.

TC: Total cholesterol.

LDL: Low density lipoprotein cholesterol.

HDL: High density lipoproteincholesterol.

TG: Triglyceride.

FBG: Fasting blood glucose.

Trop T: Troponin T.

CK: Creatinine kinase.

LVEF: Left ventricular ejection fraction.

### Hospital medications

Medication use at the time of admission is listed in Table 4. Aspirin, adenosine diphosphate antagonist, β-blockers, angiotensin-converting enzyme inhibitors, and statins were commonly prescribed to all ACS patients. The use of antiplatelet therapies, such as aspirin, was similar among all ethnic groups; more than 94% of patients in each group were taking aspirin at baseline. Malays received significantly lower proportion of low molecular weight heparin (LMWH) than other ethnic groups (p = 0.000).

**Table 4:** Medication use by ethnicity

**Medication use**	**Ethnic group**				**χ**^2**^	**df**	**P**^#^
	**Malays**	**Chinese**	**Indians**	Others*			
**Antiplatelets**							
Aspirin (%)	6092 (94.4)	2769 (94.1)	2851 (94.2)	649 (95.6)	2.6	3	0.454
Other antiplatelets (%)	4312 (68.5)	2222 (77.0)	2178 (73.0)	440 (73.0)	75.6	3	0.000
**Anticoagulants**							
Heparin (%)	907 (15.0)	202 (7.3)	281 (9.7)	55 (9.8)	128.9	3	0.000
LMWH (%)	3340 (54.1)	1882 (66.2)	2033 (68.6)	435 (68.5)	282.1	3	0.000
**Antihypertensives**							
β-blockers (%)	4238 (67.6)	2052 (71.2)	2067 (69.5)	418 (68.9)	12.3	3	0.060
ACEI (%)	3899 (62.4)	1666 (58.4)	1814 (61.1)	337 (55.9)	19.8	3	0.000
ARB (%)	468 (7.8)	242 (8.7)	304 (10.5)	59 (10.4)	20.3	3	0.000
Diuretics (%)	2016 (32.7)	798 (28.3)	888 (30.2)	196 (33.2)	19.8	3	0.000
CCB (%)	895 (14.8)	409 (14.7)	509 (17.4)	82 (14.5)	12.1	3	0.007
**Anti-diabetic agents**							
OHA (%)	1599 (26.1)	712 (25.4)	1157 (39.3)	112 (19.8)	217.9	3	0.000
Insulin (%)	1522 (24.7)	586 (20.8)	978 (33.1)	104 (18.2)	140.7	3	0.000
**Statins**							
Statins (%)	5833 (91.0)	2700 (92.1)	2737 (90.8)	646 (95.1)	16.6	3	0.001

Categorical variables are expressed as number (%).

*Others: Indigenous (Orang Asli), Kadazan, Melanau, Murut, Bajau, Bidayuh, Iban (minor ethnic groups) and other non-Malaysians.

**Pearson Chi-Square.

^#^P value.

LMWH: Low Molecular Weight Heparin.

ACEI: Angiotensin Converting Enzyme Inhibitors.

ARB: Angiotensin Receptor Blockers.

CCB: Calcium Channel Blockers.

OHA: Oral Hypoglycemic Agent.

### Invasive therapeutic procedures

Majority of patients in the registry were recorded in centers with on-site cardiac catheterization facilities and teaching hospitals (Figure 1). For ACS patients, utilization rates for elective and emergency percutaneous coronary intervention (PCI) (13.1-16.5%) and coronary artery bypass graft (CABG) (0.7-1.8%) were generally low among all ethnic groups (Table 5). The overall rates of invasive therapeutic procedures such as PCI and CABG were slightly different across the ethnic groups. Indians and Others (16.5%, 16.5%) were likely to receive PCI than Chinese and Malays (16.0%, 13.1%) (p = 0 · 000). CABG rate was slightly higher in Others (1.8%) compared to Malays (1.4%), Chinese (1.3%) and Indians (0.7%), respectively (p = 0.006). Left anterior descending (LAD) artery was the commonest culprit artery involved in ACS among all ethnic groups followed by right coronary artery (RCA) and left circumflex (LCX) artery. However, the difference across the ethnic groups was not significant (p = 0.359).

**Table 5:** Invasive procedures and culprit arteries by ethnicity

**Invasive procedures**	**Ethnic group**				**χ**^**2‡**^	**df**	**P**^#^
	**Malays**	**Chinese**	**Indians**	Others*			
PCI (%)	874(13.1)	490(16.0)	518(16.5)	119(16.5)	27.2	3	0.000
CABG (%)	95(1.4)	41(1.3)	21(0.7)	13(1.8)	12.3	3	0.006
LMA involvement (%)	184(2.8)	82(2.7)	84(2.7)	30(4.2)	5.3	3	0.150
*Culprit Artery*							
LAD (%)	591(56.3)	314(56.3)	339(58.0)	81(59.6)	13.1	12	0.359
RCA (%)	318(30.3)	172(30.8)	147(25.2)	37(27.2)			
LCX (%)	103(9.8)	53(9.5)	70(12.0)	15(11.0)			
LMA (%)	18(1.7)	8(1.4)	8(1.4)	2(1.5)			
Bypass graft (%)	20(1.9)	11(2.0)	20(3.4)	1(0.7)			

Categorical variables are expressed as number (%).

*Others: Indigenous (Orang Asli), Kadazan, Melanau, Murut, Bajau, Bidayuh, Iban (minor ethnic groups) and other non-Malaysians.

^‡^Pearson Chi-Square.

^#^P value.

PCI: Percutaneous Coronary Intervention.

CABG: Coronary Artery Bypass Graft.

LMA: Left Main Artery.

LAD: Left Anterior Descending artery.

LCX: Left Circumflex artery.

RCA: Right Coronary Artery.

### Treatment of STEMI

Table 6 shows the reperfusion strategy for STEMI including subgroup analysis for the fibrinolytic group, door-to-needle time and door-to-balloon time. Majority of STEMI patients (≥80%) received some form of reperfusion therapy either by primary PCI or fibrinolysis. Fibrinolysis appeared to be the dominant treatment options (≥70%) for all ethnic groups. Overall, significant higher proportion of Malays (78.1%) received reperfusion therapy either by primary PCI or fibrinolysis as compared with other ethnic groups (p = 0.000). Significant higher proportion of Chinese (9.0%) received primary PCI, at the same time, no reperfusion (21.5%) was higher in Chinese than other ethnic groups (P = 0.000).

**Table 6:** Treatment of STEMI by ethnicity

**Treatment of STEMI**	**Ethnic Group**				**χ**^**2‡**^	**df**^**#**^	**p**^**§**^
	**Malays**	**Chinese**	**Indians**	Others^*^			
	**No. (%)**	**No. (%)**	**No. (%)**	**No. (%)**			
Primary PCI	188 (5.6)	111 (9.0)	93 (7.9)	30 (7.4)	40.7	6	0.000
Fibrinolysis	2607 (78.1)	858 (69.5)	878 (74.3)	307 (76.2)			
No reperfusion^+^	544 (16.3)	265 (21.5)	211 (17.9)	66 (16.4)			
Total	3339 (100.0)	1234 (100.0)	1182 (100.0)	403 (100.0)			
Fibrinolysis subgroup							
Type of fibrinolytic drug used							
Streptokinase	1924(97.1)	671(98.0)	661(96.2)	238(97.9)	4.3	3	0.226
Other fibrinolytic drugs	58(2.9)	14(2.0)	26(3.8)	5(2.1)			
Door-to-needle time within 30 min	559(8.4)	181(5.9)	217(6.9)	54(7.5)	20.3	3	0.000
Door-to-needle time (min)†	50(27–100)	55(30–101)	49(24–102)	59(30–116)	10.7	3	0.013^**¥**^ (0.01, 0.02)^**¥**^
Door-to-balloon time (min)†	112(78–168)	109(73–156)	96(71–165)	81(48–124)	10.4	3	0.018^**¥**^ (0.01,0.02)^**¥**^

^*^Others: Indigenous (Orang Asli), Kadazan, Melanau, Murut, Bajau, Bidayuh, Iban (minor ethnic groups) and other non-Malaysians.

^†^Median (IQR).

^‡^Pearson Chi-Square.

^#^Degree of freedom.

^§^P value.

^+^Reason for no reperfusion includes refusal, missed fibrinolysis, and contraindication.

^**¥**^Kruskal-Wallis test with Monte Carlo level of significance (99% CI).

In the subset analysis for fibrinolysis, a higher proportion of Malays (8.4%) managed to achieve less than 30 minutes of door-to-needle time compared to other ethnic groups (P = 0.000) as recommended by Clinical Practice Guidelines [25,28]. Others reported higher door-to-needle time of 59 (IQR: 30–116) min compared to other ethnic groups (p = 0.013). On the other hand, in primary PCI, Others had the shortest door-to-balloon time of 81 (IQR: 48–124) min compared to other ethnic groups (p = 0.018). Overall, it is important to point out that we found a low percentage of patients achieving less than 30 minutes of door-to-needle time among all ethnic groups. Furthermore, door-to-needle and door-to-balloon time were longer than those recommended [25,28].

### In-hospital clinical outcomes

Indians reported significantly higher coronary care unit (CCU) days compared to Malays, Chinese and Others (3.6, 3.5, 3.2, and 2.5, respectively; p = 0.000) (Table 7). However, for total days, Malays reported significantly higher total days of admission compared to Others, Indians and Chinese (5.0, 4.9, 4.7, and 4.5, respectively; p = 0.000). Rates of major bleeding were low (≤ 0.7%) among all ethnic groups. There was no significant difference among the ethnic groups in the major or minor bleeding complication (p = 0.150) according to the Thrombolysis in Myocardial Infarction (TIMI) criteria for bleeding [29,30].

**Table 7:** In-hospital clinical outcomes by ethnicity

**In-hospital clinical outcomes**	**Ethnic group**				**F**^**†**^	**χ**^**2**^	**df**	**P**^**§**^
	**Malays**	**Chinese**	**Indians**	Others*				
CCU Days^‡^	3.5(2.8)	3.2(2.6)	3.6(2.9)	3.2(2.5)	7.4			0.000
Total Days^‡^	5.0(3.7)	4.5(3.4)	4.7(3.5)	4.9(3.7)	11.3			0.000
Bleeding rate^#^								
Major^+^	44(0.7)	13(0.5)	11(0.4)	1(0.2)		13.3	9	0.150
Minor	138(2.3)	57(2.1)	43(1.6)	11(2.1)				
Minimal	6(0.1)	1(0.0)	1(0.0)	0(0.0)				
None	5777(96.8)	2617(97.4)	2683(98.0)	524(97.8)				
Mortality rate^*#*^								
STEMI	309(9.2)	125(10.1)	96(8.1)	30(8.2)		3.3	3	0.352
NSTEMI/UA	204(6.5)	97(5.6)	69(3.7)	15(5.4)		17.8	3	0.000
**Adjusted OR for mortality (95% CI)**^¥^								
STEMI	1	0.93 (0.74, 1.16)	0.93 (0.66, 1.47)	0.99 (0.66, 1.47)				
NSTEMI/UA	1	0.71 (0.55, 0.91)	0.57 (0.43, 0.76)	0.78 (0.45, 1.34)				

*Others: Indigenous (Orang Asli), Kadazan, Melanau, Murut, Bajau, Bidayuh, Iban (minor ethnic groups) and other non-Malaysians.

CCU: Coronary Care Unit.

^**†**^One-way ANOVA.

^‡^Mean (SD).

^*#*^Number (%).

^§^P value.

^+^TIMI (Thrombolysis In Myocardial Infarction) major bleeding involves a hemoglobin drop >5 g/dL (with or without an identified site) or intracranial hemorrhage or cardiac tamponade.

^¥^Binary multiple logistic regression; ORs adjusted for age and sex.

In-hospital-mortality rate for STEMI was higher than NSTEMI and UA among all ethnic groups. STEMI mortality rate ranges from 8.1 to 10.1% which was statistically insignificant (p = 0.35) across all ethnic groups. In-hospital mortality was also not significantly different across ethnic groups in a risk adjusted multivariable model that controlled for age and sex.

For NSTEMI/UA, there was statistically significant higher in-hospital-mortality (p = 0.000) among Malays (6.5%) compared to Chinese (5.6%), Others (5.4%) and Indians (3.7%). These results were persisting after age and sex adjustment. Therefore, Malays remained a positive predictor for in-hospital mortality as compared to Chinese and Indians in the NSTEMI/UA group irrespective of age and sex.

## Discussion

The study has shown important ethnic differences in the demographics, comorbid coronary risk factors, clinical presentation, baseline investigations, treatments and in-hospital outcomes.

### Socio-demographics and coronary risk factors

Malaysia is a multiracial South East Asian country, consisting of 28.3 million people that include 67.4% Malays, 24.6% Chinese, 7.3% Indians, and 0.7% other ethnic groups [31]. However, the percentage of Indians (majority have their origins in Southern India) captured in this registry was 23.4% which indicates an over representation of the Indian population in the NCVD.

It seems that a relatively higher proportion of Indians was being captured in the NCVD Registry. However, the causal link between ethnicity and ACS is difficult to evaluate based on cross-sectional data as the NCVD Registry consists of a well-defined population diagnosed with ACS. Therefore, based on the NCVD Registry, one cannot possibly draw any conclusion as to the higher incidence of ACS among Indians compared to other ethnic groups. The finding of over-representation of Indians in the NCVD Registry was an interesting fact that serves as a basis for a prospective cohort study to reveal the association between ethnicity and ACS. Nevertheless, people of South Asian origin such as India, Pakistan, Bangladesh, Sri Lanka and Nepal represents one of the largest ethnic groups in the world and also one of the regions with the highest burden of CVDs [32]. Studies on South Asian migrants in Western countries such as Canada, United Kingdom (UK) and the United States of America (USA) documented significantly higher risks of CVDs with higher morbidity and mortality than other ethnic groups [10,14,33,34]. Previous mortality statistics and cross-sectional surveys had confirmed that Indians have an increased risk of CAD compared to other ethnic groups [11,35]. A cardiovascular cohort study showed that ethnicity plays a major role in CAD with Indian males found to have three times the risk of CAD as compared to Malays and Chinese in Singapore [36].

The mean age at presentation was 59.1 years among all ethnic groups. In contrast to earlier registries from developed countries, such as National Registry of Myocardial Infarction (NRMI) (mean age 68 years) [37], Global Registry of Acute Coronary Event (GRACE) registry (53% aged ≥ 65 years) [38,39] and the Euro Heart Survey I (EHS-ACS-I) (mean age 63.4 years) [40,41], our cohort was characterized by a younger age at presentation. The mean age was similar to the CREATE registry (58 years) [42] and Saudi Project for Assessment of Coronary Events (SPACE) registry (57 years) [43] that were conducted in India and Saudi Arabia, respectively. The age at presentation discrepancy observed between the European and Asian Registries should be investigated further to establish risk factors that possibly contributed toward the age difference.

Similar to ACS registries world-wide, the predominant sex was male (more than two thirds) among all ethnic groups. There were substantial differences between male and female in the presentation, diagnosis, management and outcome of CADs [44-47]. Most clinical trials have enrolled primarily men and the women have been under-represented in CAD clinical trials; further research involving larger female cohorts is required. Further, at participating centers, a lower proportion of women were found to have ischemia during the course of routine clinical care, and screening tests for ischemia were less predictive of CAD in women than those in men [48].

The patients’ risk factor profiles differed significantly among the ethnic groups. For example, Malays showed significantly highest mean BMI. Chinese patients had the highest proportion of hypertension and hyperlipidemia. On the other hand, Indians had the highest mean for WC and the highest proportion for DM and family history of premature CAD. Others had significantly highest proportion of smokers. The WC recorded among all ethnic groups was generally higher than the mean WC of Malaysians [49]. The finding of high WC among Indians was similar to those previously reported in the Malaysian National Health and Morbidity Survey III [49]. The finding of high prevalence of BMI among all ethnic groups with ACS was similar to another Malaysian prevalence study on overweight and obesity among non ACS population [50]. Higher BMI among Malays with ACS was also consistent with the national study on obesity among Malaysians according to ethnicity [51]. This explains the relationship between high BMI and higher occurrence of ACS in the general population [52]. The results suggest that a higher proportion of patients with ACS also have metabolic syndrome as compared to the general population [53,54]. DM has been found to be more common in Indians. This finding was consistent with previous multi-center registries and studies in Singapore [55], Trinidad [35], Fiji [56], USA [57] and UK [33,58]. A study in Canada by the Study of Health Assessment and Risk in Ethnic Groups (SHARE) investigators also found that Indians had more plasma glucose and lipid abnormalities compared to Europeans and Chinese [16]. Indians also have higher past history of CVDs and family history of MI compared to other ethnic groups [57]. Generally, our patients had high prevalence of coronary risk factors at presentation among all ethnic groups. These findings were similar to an earlier publication using NCVD [59], Singapore population [60], Iranian population [61], Saudi Arabia population [62], and in developing countries [63].

### ACS

The proportion of STEMI among all ethnic groups (38.8–58.3%) was higher compared to earlier ACS registries in developed countries: GRACE (30.0%) [38], EHS-ACS-I (42.3%) [40], NRMI (40.0%) [64], Euro Heart Survey II (EHS-ACS-II) (34.5%) [65] and Canadian Acute Coronary Syndrome Registry (27.7%) [66]. Higher proportion of STEMI compared to NSTEMI and UA in NCVD was similar to those in the CREATE registry (47.3–71.4%) [42]. In the CREATE registry, poorer socio-economic group of patients recorded a higher proportion of STEMI in India. This finding could possibly explain why Others (indigenous ethnic groups), who generally belong to the lower socioeconomic status, recorded a higher proportion of STEMI.

### Lipid profile, fasting blood glucose

Our findings on serum lipid were consistent with previous studies on Indian populations living in UK and USA. Generally, Indians have lower HDL cholesterol than whites or Afro-Caribbean populations but do not have higher total or LDL cholesterol than other races [67-69].

### Hospital medications

Results indicated high use of Aspirin, Beta-blockers, LMWH and Statins among all ethnic groups was in line with the Clinical Practice Guidelines [26,28,70] and comparable to those in developed countries. Religious practice could be a factor to explain the lower use of LMWH (containing porcine-related material) among Malays as the overwhelming majorities are Muslims compared to other ethnic groups.

### Invasive therapeutic procedures and culprit artery

On invasive therapeutic procedures, the rate of PCI and CABG in our registry was lower than those reported in developed countries [38,40,65,66]. Our results showed disparities existed in the use of medications, PCI and CABG among ethnic groups. The finding of LAD artery as the most common culprit artery was similar to those of Yadav et al. [71] and Deshpandey and Dixit [72].

### Treatment of STEMI

In STEMI, timely delivery of reperfusion therapy can reduce mortality; therefore, guidelines recommend fibrinolysis within 30 min (door-to-needle time) and primary PCI within 90 minutes (door-to-balloon-time) [25,28]. Primary PCI has been proven better than fibrinolysis in treating STEMI [73,74] and timely administration of fibrinolytic therapy significantly reduces mortality [75]. In STEMI, despite majority of patients who received reperfusion therapy, the rate of primary PCI was lower and the use of fibrinolytics was higher among all ethnic groups as compared to ACS registries in developed countries such as GRACE [38], EHS-ACS-I [40] and EHS-ACS-II [65]. In the fibrinolysis subgroup, door-to-needle time among all ethnic groups was longer than those recommended by the American and European Guidelines [25,28] and the proportion of patients achieving less than 30 minutes of door-to-needle time was low. This is one of the main concerns of our study that illustrated suboptimal care in treating STEMI. Streptokinase, the less-fibrin specific (less effective) fibrinolytic agent as compared with fibrin-selective fibrinolytic agents [76] was commonly used among all ethnic groups. Higher proportion of Chinese received less reperfusion therapy than other ethnic groups; however, the reasons for these discrepancies were unclear.

In the implementation of evidence-based reperfusion strategies, doctors have to make the best decision to allocate limited resources to patients who are at highest risk and hope to obtain the largest benefit. Many studies have found differences in the delivery of cardiac care and reperfusion procedure among different ethnic groups in ACS [77-79]. Earlier studies of ethnic variation in the treatment and outcome of ACS in USA showed that non-whites had longer door-to-needle time in the treatment of AMI [80] and were less likely to undergo invasive cardiac procedures [81,82]. However, this finding was controversial in other clinical trials [83]. Research has shown that by implementing a national quality improvement program, it is possible to reduce or eliminate the differences in care by ethnicity [84]. Considerably more effort and resources will be granted to improve the reperfusion strategies in developing countries like Malaysia for all patients regardless of their ethnic origin. Adherence to clinical practice guidelines has shown to improve quality of care and associated with significant reduction in in-hospital mortality rates [85].

### In-hospital clinical outcomes

In developed countries, STEMI mortality rates were reported as 4.3–4.4% in NRMI [86], 7.0% in the GRACE Registry [38], 7.0% in the EHS-ACS-I [40] and 2.4% in the Canadian Acute Coronary Syndrome Registry [66]. In the NCVD, higher STEMI mortality rate (8.1–10.1%) in comparison with developed countries could be explained by lower use of primary PCI, higher use of less effective fibrinolytic agent (Streptokinase) and delay in door-to-needle time among all ethnic groups. In addition, LAD artery involvement as the main culprit vessel (>56%) among all ethnic groups could have contributed towards a less favorable outcome as reported earlier by Thanavaro et al. [87].

Interestingly, despite the fact that disparities exist in risk factors, clinical presentation, medical treatments and invasive management, there was no statistically significant difference in STEMI mortality among all ethnic groups. However, there are limitations in the analysis of mortality outcome comparing different ethnic groups and we advise caution in its interpretation. Firstly, it was an in-hospital mortality of STEMI and NSTEMI/UA of both genders and all age groups. As for ACS, many studies had shown higher mortality in women [88,89] and older patients [39,41,90]. Hence, the mortality outcome analyzed in this method may have over or underestimated the differences between age groups and across genders. Secondly, we used in-hospital all-cause mortality as an end point in order to minimize event misclassification. Results for cardiac-specific mortality may be different for present results. Caution should, therefore, be observed in comparing results with those from other national registries or clinical trials.

On the basis of the risk factor profiles across all ethnic groups, it would seem unreasonable for Malays to have higher NSTEMI and UA mortality. It would seem that higher mortality in Malays can be partly explained by the higher proportion of Malays receiving lower rates of PCI compared to other ethnic groups. Other possible explanations include greater cigarette consumption and higher BMI among Malays.

Earlier observational studies locally and elsewhere have shown a higher proportion of deaths in Indians with IHD compared to other ethnic groups [11,35,91]. The finding of lower in-hospital mortality in NSTEMI/UA among Indians in our study contradicted with previous studies for unexplained reasons.

There were evidences that bleeding risk in ACS differs in different type of ACS, reperfusion therapy and ethnic groups [92]. Registry data [92], prospective study [93] and randomized controlled trials (RCT) [94] of developed countries in ACS patients reported that the major bleeding risk was between 4.6–10.9%. Compared to developed countries, major bleeding in NCVD was generally low among all ethnic groups (0.2–0.7%) and this finding was similar to those in the ACS Registry in India (0.2–0.3%) (CREATE) and Middle Eastern countries (0.83%) [95]. The finding of a lower risk of major bleeding is intriguing and possibly could be explained by the lower use of invasive cardiac procedures among all ethnic groups as compared to those in developed countries.

### Strengths and limitations

The main strength of this study is the collection of data from multi centers to represent a complete and unselected group of patients in a real-world setting. Unlike restricted populations recruited in RCTs which tend to exclude high risk patients, the NCVD collects data on the full spectrum of ACS patients from a nationwide perspective. We gain insight into ACS patients that were not included in RCTs. We use a standard method across all ethnic groups in collecting data to avoid bias.

Several limitations of our study should be noted. Firstly, misclassification of ethnicity can occur as ethnicity was self-reported and mutually-exclusive. Secondly, the possibility of selection bias despite our attempt to include hospitals in different regions of the country. Many private hospitals with significant number of ACS admissions possibly with a different ethnic distribution did not participate in this registry. Ethnic minorities living in remote areas had difficulty accessing the health facilities and therefore could have been under-represented in this study. Thirdly, there may have been underreporting of risk factors such as cigarette smoking and past medical history as these measures were self-reported and this may subject to bias.

Furthermore, statistical differences are frequently observed in a large number of subjects but may not be clinically meaningful. In studies with large patient enrolment, small differences between groups will be highly significant by conventional use of the *p* values, and the clinical importance of these differences can only be judged with clinical insight.

## Conclusions

There are two major implications of our study. First, we have shown interesting facts about the differences and similarities of demographic, risk profile, ACS stratum, treatment and outcome among different ethnic groups. Second, our study establishes a critical understanding of disease spectrum, drug usage, invasive procedures, clinical outcomes, and the overall quality of care provided to ACS patients in Malaysia. In addition, the NCVD provides insights and forms the basis for clinical investigators to design future clinical trials. The NCVD also enables clinicians and health care administrators to compare disease-specific patterns, management and outcomes which might lead to changes in national health priorities or strategies of disease management.

## Abbreviations

ACS: Acute coronary syndrome; BMI: Body mass index; CABG: Coronary artery bypass graft; CCU: Coronary care unit; CREATE: Treatment and outcomes of ACS in India; CVD: Cardiovascular disease; DM: Diabetes mellitus; GRACE: Global Registry of Acute Coronary Events; IHD: Ischemic heart disease; LAD: Left anterior descending; LCX: Left circumflex; LM: Left main; LMWH: Low molecular weight heparin; LVEF: Left ventricular ejection fraction; MOH: Ministry of Health; NCVD: National Cardiovascular Disease Database Registry; NRMI: National Registry of Myocardial Infarction; NSTEMI: Non ST-segment elevation myocardial infarction; PCI: Percutaneous coronary intervention; RCA: Right coronary artery; RCT: Randomized controlled trial; SPACE: Saudi Project for Assessment of Coronary Events; STEMI: ST-elevation myocardial infarction; UA: Unstable angina; WHO: World Health Organization; TIMI: Thrombolysis in myocardial infarction; WC: Waist circumference.

## Competing interests

Both authors declare that they have no competing interests.

## Authors’ contributions

LHT and RBN collated and analyzed the data. LHT prepared the first draft of the paper and RBN vetted the final manuscript. Both authors interpreted the results, revised the paper, and approved the final version.

## Pre-publication history

The pre-publication history for this paper can be accessed here:

http://www.biomedcentral.com/1471-2261/13/97/prepub
